# Associations between sleep duration, physical activity, and cognitive impairment in older adults—empirical analysis based on CHARLS data

**DOI:** 10.3389/fpubh.2025.1589606

**Published:** 2025-06-02

**Authors:** Miao Ma, Feng-Wei Dong, Jin-Yan Lan

**Affiliations:** ^1^Department of Physical Education, Yuncheng University, Yuncheng, China; ^2^Martial Arts Academy, Wuhan Sports University, Wuhan, Hubei, China

**Keywords:** older adults population, sleep duration, physical activity, cognitive impairment, joint effects

## Abstract

**Objective:**

To systematically analyze the independent and combined effects of abnormal sleep duration and insufficient physical activity on the risk of cognitive impairment in older Chinese adults, to elucidate the regulatory mechanisms of physical activity in the sleep-cognition relationship, and to provide a robust scientific foundation for the development of multidimensional cognitive health intervention strategies.

**Methods:**

A total of 5,184 older adults (aged 60 years and above) were selected for analysis from the 2020 China Health and Retirement Longitudinal study (CHARLS) data. The Mini-Mental State Examination (MMSE) and the International Physical Activity Questionnaire-Short Form (IPAQ-SF) were utilized to assess cognitive function and physical activity levels, respectively. Multifactorial logistic regression models were employed to analyze the independent and joint effects of sleep duration and physical activity on cognitive impairment, as well as to investigate the moderating role of physical activity.

**Results:**

Both short sleep duration (less than 6 h per night, OR = 1.274, 95%CI: 1.099 ~ 1.477) and long sleep duration (more than 8 h per night, OR = 1.228, 95%CI: 1.044 ~ 1.445) significantly increased the risk of cognitive impairment. Additionally, low-physical activity levels (less than 600 MET-min/week, OR = 1.436, 95%CI: 1.091 ~ 1.890) were also associated with a heightened risk of cognitive impairment. The interaction between sleep duration and physical activity was significant; specifically, short sleep combined with low-physical activity (OR = 2.196, 95% CI: 1.385 ~ 3.484) and long sleep with low-physical activity (OR = 1.273, 95%CI: 1.078 ~ 1.503) significantly elevated the risk of cognitive impairment. The prevalence of cognitive impairment was significantly different (*p* < 0.01) between the group with suitable sleep and moderate-high physical activity (24.01%) and the other combination groups.

**Conclusion:**

The results of this study support the hypothesis that sleep duration and physical activity levels may collaboratively enhance cognitive health through various physiological mechanisms. Furthermore, the findings suggest that a combination of sleep and physical activity interventions could be effective in preventing cognitive decline in the older adults population. However, due to the inherent limitations of the cross-sectional design, the findings reflect only statistical associations rather than causal relationships between the variables. Future studies should employ prospective designs and objective measures to investigate the causal relationships and underlying mechanisms in greater depth.

## 1 Introduction

With the accelerated aging of the global population, Chinese society is experiencing a significant demographic transition. According to the latest report from the World Health Organization (WHO), the older adults population in China, aged 60 years and older, has surpassed 280 million, accounting for over 20% of the total population. Consequently, the issue of cognitive impairment among the older adults is becoming increasingly severe. Epidemiological survey data indicate that the prevalence of cognitive impairment among Chinese individuals aged 60 and above has reached 22.24% (1), which is significantly higher than the global average. Cognitive impairment not only leads to a comprehensive decline in memory, learning, and executive function among the older adults but can also progress to irreversible neurodegenerative diseases such as Alzheimer’s disease and dementia with Lewy bodies (2). This disease burden not only severely impacts the quality of life for patients but also imposes substantial economic pressure on families and society (3). Given that there are currently no effective means to eradicate cognitive impairment, exploring early prevention and intervention strategies based on modifiable lifestyle factors has become a focal point of research in the fields of geriatrics and public health worldwide.

Among the various lifestyle factors that influence cognitive health, the roles of sleep and physical activity have garnered significant attention. The duration, continuity, and quality of sleep, as essential physiological processes for maintaining brain homeostasis, are closely linked to cognitive function (4, 5). Numerous longitudinal studies have confirmed that consistently short sleep (less than 6 h per night) leads to abnormal deposition of *β*-amyloid and tau proteins, which accelerates neuroinflammatory responses and subsequently triggers cognitive decline (6, 7). Conversely, prolonged sleep (more than 8 h per night) may indicate underlying cardiovascular disease, metabolic disorders, and other health issues, all of which are similarly associated with an increased risk of cognitive impairment (8). The protective effect of physical activity on cognitive health has been extensively validated. A systematic review conducted by the International Association of Geriatric Psychiatry (IAGP) indicated that engaging in regular moderate- to high-intensity exercise can reduce the risk of cognitive impairment by 30–40%. This reduction is attributed to the promotion of brain-derived neurotrophic factor (BDNF) secretion, improved cerebral blood perfusion, and enhanced neural plasticity (9). Additionally, the Chinese CHARLS cohort study revealed that the risk of cognitive impairment among physically inactive older adults was 33% higher compared to those who were adequately active (10). However, most current studies have concentrated on the independent effects of individual factors, leaving a gap in research regarding the mechanisms underlying the combined effects of sleep and physical activity (11). While a few studies have attempted to explore the interaction between these two factors, their small sample sizes and the lack of systematic validation of the moderating effect of physical activity on the sleep-cognition relationship limit the development of effective intervention strategies.

There is a complex interrelationship between sleep and physical activity (12). A substantial body of evidence suggests that physical activity can indirectly influence cognitive function by enhancing sleep quality, regulating circadian rhythms, and reducing sleep-related metabolic stress (13). Furthermore, the coexistence of sleep deprivation and physical inactivity may have a compounded effect on various mechanisms, further increasing the risk of cognitive decline (14). Based on this, the present study proposes the following hypotheses: First, both abnormal sleep duration (less than 6 h per night or more than 8 h per night) and low-physical activity levels (less than 600 MET-minutes per week) are independent risk factors for cognitive impairment in older Chinese adults. Second, there is a significant synergistic effect between abnormal sleep duration and low-physical activity levels, and combined exposure to both factors will significantly elevate the risk of cognitive impairment. Third, physical activity moderates the relationship between sleep duration and cognitive impairment.

The primary objectives of this study are as follows: First, utilizing the 2020 CHARLS data, we aim to systematically examine the independent effects of abnormal sleep duration and insufficient physical activity on the risk of cognitive impairment among older adults in China, employing rigorous statistical methods. Second, we will analyze the combined effects of sleep duration and physical activity on cognitive impairment and validate the synergistic relationship between the two factors. Third, we seek to clarify the moderating role of physical activity in the relationship between sleep and cognition. By adopting a multifactorial perspective, we aim not only to enhance the understanding of the intrinsic mechanisms linking sleep duration and physical activity levels to cognitive health in the older adults but also to provide targeted, actionable scientific evidence and policy recommendations for establishing early screening indicators and developing comprehensive intervention strategies for cognitive disorders among older adults in China.

## 2 Materials and methods

### 2.1 Data sources

The data utilized were obtained from the China Health and Retirement Longitudinal study (CHARLS), a nationwide longitudinal survey of middle-aged and older adults individuals aged 45 years and older. Conducted every 2–3 years since 2011 by the National Development Research Institute of Peking University, CHARLS boasts a high degree of national representativeness. The baseline survey encompassed 450 villages across 150 counties in 28 provinces (including autonomous regions and municipalities directly under the central government) throughout the country. The survey collected information on physical activity levels, cognitive function, self-rated health status, and demographic variables (15), providing robust data to explore the combined effects of sleep duration and physical activity on the risk of cognitive impairment in the older adults. All data collection processes received approval from the Biomedical Ethics Review Board of Peking University (approval number: IRB00001052-11015), and written informed consent was obtained from all participants.

**Figure 1:**
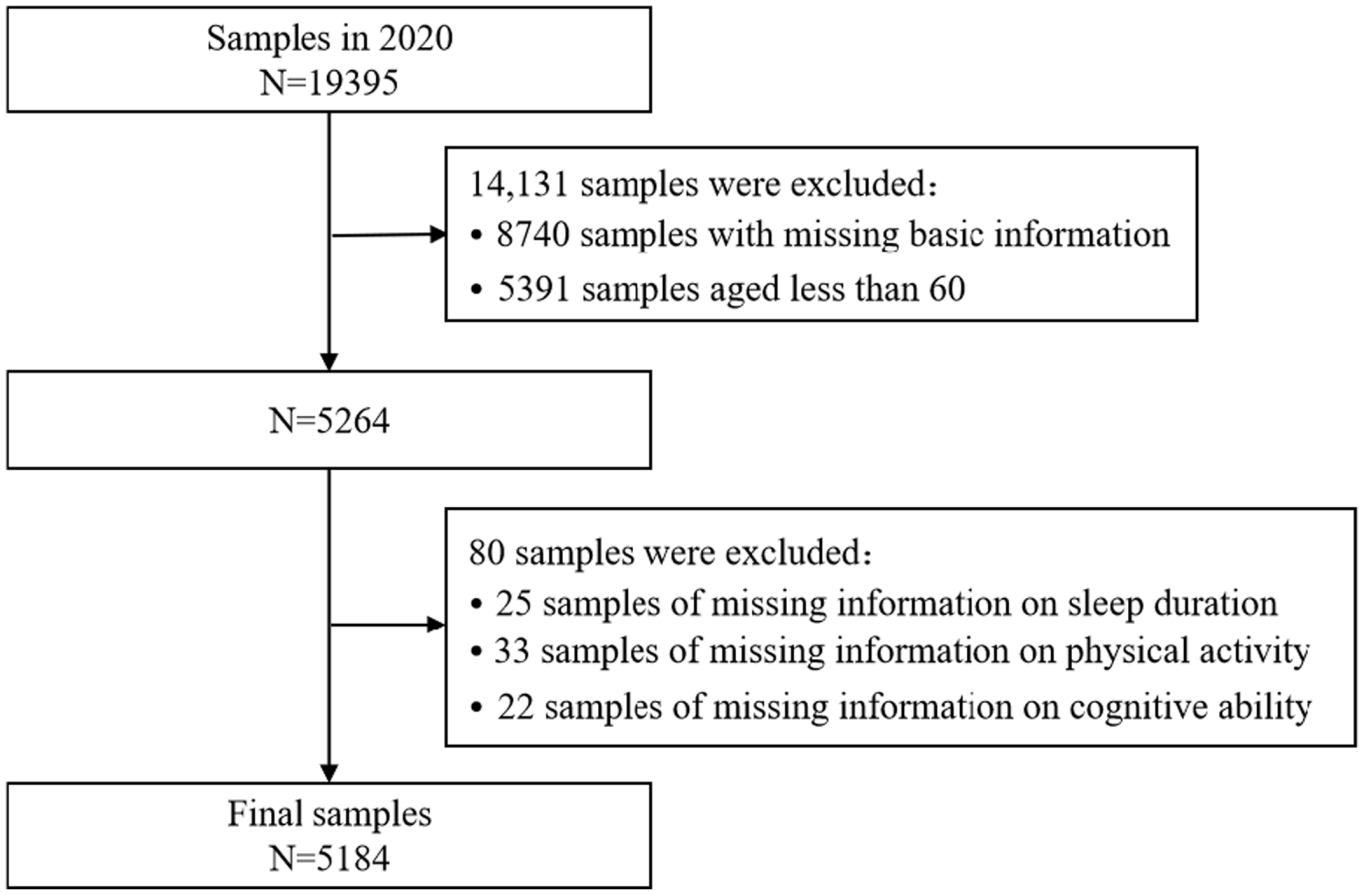
Sample screening flowchart.

Samples were systematically screened using the 2020 CHARLS data. The original dataset comprised 19,395 samples, and during the screening process, ineligible samples were gradually excluded based on the study’s objectives and the integrity of the variables. Initially, 8,740 samples were excluded for lacking essential basic information (e.g., gender, place of residence), and 5,391 samples were excluded for being younger than 60 years of age, thereby focusing the sample on the target group of older adults (≥60 years), which resulted in a remaining sample size of 5,264. Subsequently, the sample was further refined by excluding 25 samples with missing information on sleep duration, 33 samples with missing data on physical activity, and 22 samples with missing information on cognitive ability. The core variables of interest were sleep duration, physical activity level, and cognitive function, leading to a final sample of 5,184 older adults who met the analysis criteria. This sample size ensures that the study possesses strong statistical test efficacy and can effectively detect potential associations between sleep duration, physical activity, and cognitive impairment. The specific exclusion criteria and screening process are illustrated in Figure 1.

### 2.2 Variable selection

#### 2.2.1 Assessment of cognitive function

Cognitive impairment, the primary outcome variable of this study, was assessed using the Chinese version of the Mini-Mental State Examination (MMSE) (16). This tool was selected to calculate overall scores across four dimensions: date cognition, recall ability, computational ability, and drawing ability, in order to evaluate the cognitive levels of the interviewees. The scoring for each dimension is as follows: date cognition (0–5), recall ability (0–20), computational ability (0–5), and drawing ability (0–1). The total score ranges from 0 to 31, with higher scores indicating better cognitive ability. To accurately identify the presence of cognitive impairment, study participants were categorized based on their education levels. According to the MMSE rating criteria, cognitive impairment was identified under the following conditions: (1) for individuals with an illiterate education level, a cognitive score of less than 17; (2) for those with a primary education level, a cognitive score of less than 20; and (3) for individuals with a secondary education level or higher, a cognitive score of less than 24.

#### 2.2.2 Assessment of sleep duration

Based on the 2020 CHARLS questionnaire, study participants were asked, “In the past month, approximately how many hours did you actually fall asleep each night?” and “In the past month, how long did you usually take a nap?” These questions aimed to calculate the participants’ self-reported average length of sleep over the past month. In this study, nap durations were categorized as follows: no nap was assigned 0 h; 1–30 min was assigned 0.5 h; 31–60 min was assigned 1 h; 61–120 min was assigned 2 h; and more than 120 min was assigned 3 h, consistent with previous studies (17). The total sleep duration was defined as the sum of the duration of continuous nighttime sleep and the duration of naps. Sleep duration was classified as follows: less than 6 h was defined as short sleep, 6–8 h as suitable sleep, and more than 8 h as long sleep.

#### 2.2.3 Assessment of physical activity level

Respondents’ physical activity levels were assessed using data collected from the“Lifestyle”and “Health Behavior”sections of the CHARLS questionnaire. First, respondents were asked to review and report the amount of time they spent on various types of physical activity over the past week. The frequency of physical activity was categorized into four levels: 0 days per week, 1–2 days per week, 3–5 days per week, and 6–7 days per week. According to the criteria of the International Physical Activity Questionnaire-Short Form (IPAQ-SF), the duration of daily physical activity was categorized into five bands: 0, 10–29, 30–119, 120–239, and≥240 min. The median of each band was calculated for subsequent analyses. Second, different types of physical activity were assigned corresponding Metabolic Equivalent (MET) values to quantitatively measure the intensity of the activities. The specific values were assigned as follows: the MET value for high-intensity physical activities, such as climbing, running, and farming, was set at 8.0; for moderate-intensity activities, such as brisk walking and tai chi, the value was 4.0; and for low-intensity activities, such as leisurely walking, it was 3.3. The formula for calculating MET is as follows: coefficientt * number of dayst * minutes of physical activity per session. This formula is used to quantitatively assess an individual’s level of physical activity (18). A higher MET value indicates a greater level of physical activity. Finally, based on the total MET minutes per week, the physical activity levels of the respondents were categorized into three groups: high-intensity physical activity (3,000 METs/week), moderate-intensity physical activity (600–3,000 METs/week), and low-intensity physical activity (≤600 METs/week).

#### 2.2.4 Selection of covariates

Considering that various factors may influence cognitive impairment, age, gender, place of residence, marital status, sleep quality, physical activity level, type of chronic disease, education level, smoking status, and alcohol consumption were selected as covariates for this study. Age was categorized into three groups: 60–69, 70–79, and 80 years or older. Gender was coded as 1 for male and 0 for female. Place of residence was coded as 1 for urban areas and 0 for rural areas. Marital status was assigned a value of 1 for married individuals and a value of 0 for all other statuses. The classification of chronic diseases was determined by the physician’s diagnosis. No chronic disease was assigned a value of 0, one chronic disease received a value of 1, two chronic diseases were assigned a value of 2, and three or more chronic diseases were assigned a value of 3. Education levels were categorized into three groups: elementary school and below, junior high school, and senior high school and above, with corresponding values of 1, 2, and 3, respectively. Smoking and drinking statuses were assigned values of 1 for and 0 for The selection of these covariates was informed by previous research findings and aimed to comprehensively control for potential confounding factors in the relationship between sleep duration, physical activity, and cognitive impairment. The specific variable selection is presented in Table 1.

**Table 1:** Selection of variables.

Variable types	Variable name	Variable symbol	Description of assignment
Explained variables	Cognitive function	MMSE	Presence of cognitive impairment is assigned a value of 1, otherwise 0
Explanatory variables	Length of sleep	Sleep	Short sleep is assigned 1, suitable sleep is assigned 2, long sleep is assigned 3
	Physical activity level	Exercise	Low-physical activity is assigned 1, moderate-high physical activity is assigned 2
Covariate	Age	Age	60–69 assigns value 1, 70–79 assigns value 2, ≥80 assigns value 3
	Gender	Gender	Assign a value of 1 to males and a value of 0 to females
	Place of residence	Urban	Urban is assigned a value of 1, rural is assigned a value of 0
	Marital status	Marry	Married is assigned a value of 1, others are assigned a value of 0
	Types of chronic diseases	Disease	3 or more chronic diseases are assigned a value of 3, 2 chronic diseases are assigned a value of 2, 1 chronic disease is assigned a value of 1, and no chronic disease is assigned a value of 0
	Educational level	Edu	Elementary school and below is assigned a value of 1, middle school is assigned a value of 2, and high school and above is assigned a value of 3
	Smoking history	Smoke	Yes is assigned 1, no is assigned 0
	Drinking history	Drink	Yes is assigned 1, no is assigned 0

### 2.3 Statistical analysis

Data processing and analysis were conducted using Stata 16.0 statistical software. In the data preprocessing stage, missing values for each variable were thoroughly examined, and samples with incomplete data on sleep duration or physical activity were excluded. Simultaneously, the mean, standard deviation, minimum, and maximum values were calculated for continuous variables, and descriptive statistics were employed to analyze data distribution characteristics to determine their suitability for subsequent analysis. Next, the association between categorical variables, such as sleep duration and physical activity level, and cognitive impairment was analyzed using the chi-square test. The relationship between ordered categorical variables, such as age and education level, and cognitive impairment was explored using the *t*-test. This approach aimed to clarify the influence of ordered categorical variables on cognitive impairment by evaluating the differences in mean values between groups and to preliminarily screen for variables related to cognitive impairment. Finally, multifactor logistic regression models were employed to investigate the independent and joint effects of sleep duration and physical activity on cognitive impairment. Cognitive impairment served as the dependent variable in the model, while sleep duration (short sleep = 1, suitable sleep = 2, long sleep = 3) and physical activity level (low = 1, mederate -high = 2) were designated as the primary independent variables, controlling for covariates such as age and gender. The goodness-of-fit of the model was assessed using the Hosmer-Lemeshow test. Additionally, interaction analyses were conducted by incorporating an interaction term between sleep duration and physical activity, and multicollinearity was addressed by evaluating variance inflation factors (VIF) to exclude severe covariance. Results are presented as odds ratios (OR), 95% confidence intervals (95%CI), and *p*-values, with the significance level set at *p* < 0.05.

## 3 Results

### 3.1 Basic information on cognitive impairment in older adults

Of the 5,184 older adults individuals aged 60 years and older included in this study, the age range was from 60 to 91 years. Among the participants, 56.31% were male and 43.69% were female. The prevalence of cognitive impairment among all subjects was 26.89%. The results of the χ^2^ test and t-test presented in Table 2 indicated that age, place of residence, marital status, education level, alcohol consumption, sleep duration, and physical activity level were significantly different between the cognitive impairment group and the normal group (*p* < 0.01). In contrast, gender and smoking status were not significantly associated with cognitive impairment (*p* > 0.05).

**Table 2:** Basic analysis of cognitive impairment in the older adults.

Broad category	Subcategories	Number of participants (*n* = 5,184)	Cognitively normal (*n* = 3,790)	Cognitively impaired (*n* = 1,394)	χ^2^-value	P-value
Age	60–69	3,413	2,586	827	81.436	0.000
	70–79	1,513	1,073	440		
	≥80	258	131	127		
Gender	Male	2,265	1,670	595	0.789	0.374
	Female	2,919	2,120	799		
Place of residence	Urban	2,593	1,806	787	31.602	0.000
	Rural	2,591	1,984	607		
Marital status	Married	4,257	3,162	1,095	16.522	0.000
	Others	927	628	299		
Types of chronic diseases	No chronic disease	713	538	175	6.161	0.104
	1 chronic disease	1,034	754	280		
	2 chronic diseases	1,092	770	322		
	3 chronic diseases	2,345	1,728	617		
Educational level	Elementary and below	3,219	2,419	800	64.420	0.000
	Middle school	1,165	747	418		
	High school and above	800	624	176		
Smoking history	No	2,602	1,919	683	1.093	0.296
	Yes	2,582	1,871	711		
Drinking history	No	3,240	2,325	915	8.013	0.005
	Yes	1944	1,465	479		

### 3.2 Multicollinearity test

The covariance between the variables was evaluated by calculating the variance inflation factor (VIF). The results presented in Table 3 indicate that the VIF values for all variables were below the critical threshold of 10, with the highest value for gender being 2.24. Additionally, the average VIF value was 1.31, suggesting that the variables were selected appropriately and that there were no significant issues with covariance.

**Table 3:** Results of multiple covariance test.

Variable name	Variance inflation factor	1/variance inflation factor
Age	2.34	0.427803
Gender	2.10	0.476313
Urban	1.21	0.827011
Marry	1.11	0.90215
Disease	1.10	0.906323
Edu	1.09	0.919732
Smoke	1.09	0.921094
Drink	1.03	0.966268
Mean variance inflation factor	1.31	

### 3.3 Independent effects of sleep duration and physical activity on cognitive impairment

**Figure 2:**
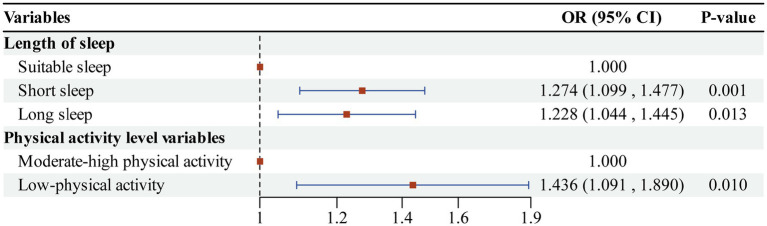
Logistic regression results of factors affecting cognitive impairment.

We analyzed cognitive impairment as the dependent variable, with sleep duration and physical activity level serving as independent variables, using multifactor logistic regression models. During model construction, appropriate sleep duration and moderate-high physical activity were designated as control groups, while covariate factors and other potential confounders were controlled for separately. The results of the regression analysis presented in Figure 2 indicated that (1) the independent effect of sleep duration: the risk of cognitive impairment was significantly higher in the short sleep group (less than 6 h per night) compared to the suitable sleep group (OR = 1.274, 95%CI: 1.099 ~ 1.477, *p* < 0.01). Additionally, the risk of cognitive impairment was also significantly higher in the long sleep group (more than 8 h per night) compared to the suitable sleep group (OR = 1.228, 95%CI: 1.044 ~ 1.445, *p* < 0.05). (2) Regarding the independent effect of physical activity level: the risk of cognitive impairment was significantly higher in the low-physical activity group (less than 600 MET-min/week) compared to the moderate-high physical activity group (OR = 1.436, 95%CI: 1.091 ~ 1.890, p < 0.01). The first study hypothesis was confirmed; both abnormal sleep duration (less than 6 h per night or more than 8 h per night) and low-physical activity level (less than 600 MET-min/week) were identified as independent risk factors for cognitive impairment among older adults in China.

### 3.4 Joint effects of sleep duration and physical activity on cognitive impairment

**Figure 3:**
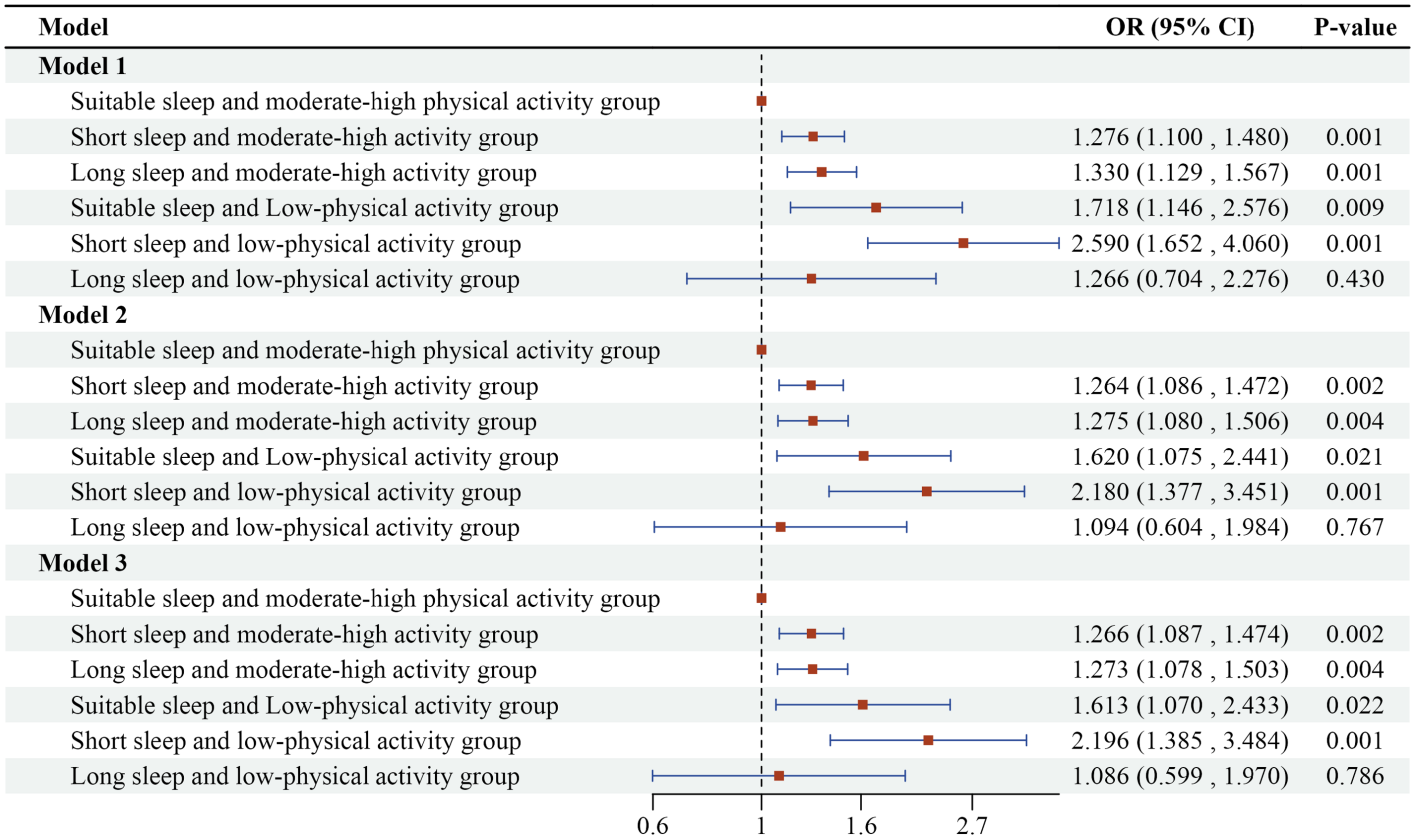
Logistic regression results of the combined effects of sleep duration and physical activity on cognitive impairment in older adults.

The interaction term between sleep duration and physical activity level was introduced into the multifactorial logistic regression model, with uncorrected variables in model 1; age, gender, education, marital status, and place of residence corrected in model 2; and smoking, alcohol consumption, and chronic disease status corrected in model 3 on the basis of model 2. The regression results in Figure 3 indicate that the interaction term was statistically significant (*p* < 0.05), suggesting that there is a significant synergistic effect between sleep duration and physical activity level, which together affect the risk of cognitive impairment. The results of Model 3 indicated a 119.6% increased risk of cognitive impairment in the short sleep and low-physical activity group (OR = 2.196, 95%CI: 1.385 ~ 3.484) compared to the suitable sleep and moderate-high physical activity group. Additionally, there was a 26.6% increased risk of cognitive impairment in the short sleep and moderate-high physical activity group (OR = 1.266, 95%CI: 1.087 ~ 1.474) compared to the suitable sleep and moderate-high physical activity group. The group with long sleep and moderate-high physical activity (OR = 1.273, 95%CI: 1.078 ~ 1.503) exhibited a 27.3% increase in the risk of cognitive impairment compared to the group with suitable sleep and moderate-high physical activity. This finding supports hypothesis two, which posits that there is a significant synergistic effect of both abnormal sleep duration and low-levels of physical activity. Combined exposure to these two factors significantly elevates the risk of developing cognitive impairment.

Further stratified analyses revealed that physical activity levels significantly moderated the relationship between sleep duration and cognitive impairment. Using the suitable sleep as a reference, low-physical activity increased the risk of cognitive impairment from 24.01% (for moderate to high physical activity) to 35.19%, representing a 46.56% increase. In the short sleep group, moderate-high physical activity also elevated the risk of cognitive impairment compared to the suitable group (28.73% vs. 24.01%); however, the increase in risk diminished from 87.42 to 19.66% when compared to the low-physical activity group (45.00%). This indicates that moderate to high-physical activity significantly mitigates the rising trend of cognitive impairment risk in scenarios of abnormal sleep duration, as illustrated in Table 4. In addition, the moderating effect was quantified in terms of risk difference: the difference in the risk of cognitive impairment between low and moderate-high physical activity was 16.27% in the short sleep group; in the long sleep group, it was 1.24%; and in the suitable sleep group, it was only 11.18%. This further confirms that the moderating effect of physical activity on the risk of cognitive impairment is more pronounced in individuals with abnormal sleep patterns. These results support Hypothesis 3, which posits that physical activity moderates the relationship between sleep duration and cognitive impairment. This suggests that a combination of sleep and physical activity interventions may be more effective in delaying cognitive decline in older adults.

**Table 4:** Interaction effect of sleep duration and physical activity level on cognitive impairment.

Groups	Cognitively normal (*n* = 3,790)	Cognitively impaired (*n* = 1,394)	Incidence of cognitive impairment (%)
Suitable sleep and moderate-high physical activity group	1,991	629	24.01
Short sleep and moderate-high physical activity group	955	385	28.73
Long sleep and moderate-high physical activity group	690	290	29.59
Suitable sleep and low-physical activity group	70	38	35.19
Short sleep and low-physical activity group	44	36	45.00
Long sleep and low-physical activity group	40	16	28.57

## 4 Discussion

Based on data from the 2020 China Health and Retirement Longitudinal study (CHARLS), this study systematically analyzed the combined effects of sleep duration and physical activity on cognitive impairment among Chinese older adults, while also exploring the moderating role of physical activity in this context. The results indicated that both sleep duration and physical activity levels significantly influenced the risk of cognitive impairment, and a notable interaction between the two was observed. This finding not only enhances the existing research on cognitive health in older adults but also provides a scientific foundation for the development of comprehensive intervention strategies.

It was found that both short sleep (less than 6 h per night) and long sleep (more than 8 h per night) significantly increased the risk of cognitive impairment, with the risk being greater in the short sleep group compared to the long sleep group. This finding corroborates numerous national and international studies and highlights unique group characteristics. In terms of international research, a large-scale cohort study conducted by the Alzheimer’s Disease Association of the United States followed over 1,000 older adults and discovered that individuals who slept less than 6 h had a 42% higher risk of cognitive impairment over a 5-year period compared to those with normal sleep duration (19). This result aligns closely with the present study’s finding that short sleep increases the risk of cognitive impairment. Conversely, a Japanese community-based study involving 3,000 older adults aged 65 and above similarly confirmed a significant association between short sleep and cognitive decline in an Asian population (20). Among the studies related to long sleep, the European Aging Consortium (2023) has demonstrated that while the increased risk of cognitive impairment in long sleep populations (those sleeping more than 8 h per night) is relatively small compared to that in short sleeping populations, this group often exhibits a higher prevalence of chronic diseases such as cardiovascular disease and diabetes. These comorbidities are strongly associated with cognitive decline (21). The present study further reveals that long sleep may impact cognitive function by reflecting underlying health issues, such as metabolic syndrome and an enhanced inflammatory response, in the older adults Chinese population (22). In contrast to other studies, this research is more nationally representative, based on a large sample analysis of CHARLS data. It refines the association between sleep duration and cognitive impairment by considering the lifestyles and health conditions of older Chinese adults, thereby complementing the existing research evidence in this area concerning the Asian older adults population. On the other hand, the positive effects of physical activity on cognitive health were reaffirmed in the present study, which provided more detailed insights compared to previous research. Consistent with the findings of Liu et al. (2024), which indicated that moderate-high intensity physical activity reduces the risk of cognitive impairment (23), this study further quantified the impact of low-physical activity (<600 MET-min/week) on cognitive impairment (OR = 1.436, 95%CI:1.091 ~ 1.890). Internationally, the Canadian Study of Health and Aging (2022) demonstrated that older adults who engaged in at least 150 min of moderate-intensity exercise per week experienced a 30% slower rate of cognitive decline compared to non-exercisers (24). Additionally, a randomized controlled trial in Australia found that long-term regular exercise increased hippocampal volume by 2 to 3% and significantly improved memory function (13). Notably, considering the life characteristics of older adults in China, this study further explored the effects of low-intensity activities, such as daily housework and traditional fitness practices, on cognitive function. This approach offers a more relevant perspective on the relationship between physical activity and cognitive health in real-life scenarios.

Another important finding of this study is the significant interaction between sleep duration and physical activity level (25), which has a compounded effect on the development of cognitive impairment. Specifically, the risk of cognitive impairment was notably higher in both the short sleep and low-physical activity group and the long sleep and low-physical activity group compared to the suitable sleep and moderate-high physical activity group. This suggests that merely adjusting sleep or physical activity levels in isolation may not be sufficient to significantly reduce the risk of cognitive impairment (26). Therefore, a combined intervention strategy that integrates both factors should be considered. This interaction may arise from the synergistic effects of multiple physiological mechanisms. From the perspective of the neuroinflammatory response, abnormalities in sleep quality and duration activate microglia in the brain, causing them to remain in a constant state of hyperactivity. This hyperactivity results in the release of large amounts of pro-inflammatory cytokines (27). These inflammatory factors can compromise the integrity of the blood–brain barrier, facilitating the entry of peripheral inflammatory substances into the brain and further exacerbating neuroinflammation. Additionally, long-term physical inactivity contributes to elevated levels of chronic inflammation in the body. Research has demonstrated that a sedentary lifestyle prompts adipose tissue to secrete more inflammatory mediators, intensifying the systemic inflammatory response (28). When sleep abnormalities coexist with low-physical activity, they create a vicious cycle at the level of neuroinflammation. Persistent neuroinflammation damages neurons, disrupts synaptic connections, affects neural signaling, and ultimately leads to impaired cognitive function (29). In terms of metabolic state in the brain, sleep deprivation disrupts the normal energy metabolism processes. During sleep, the brain engages in several critical metabolic activities, including the removal of metabolic waste and the restoration of energy reserves (30). Both short and long sleep durations can disturb this rhythm, resulting in impaired glucose metabolism and insufficient energy supply to the brain (31). Conversely, physical activity exerts a beneficial regulatory effect on intracerebral metabolism. The increased blood flow to the brain during exercise enhances the delivery of oxygen and nutrients to neurons, promotes glucose uptake and utilization, and supports normal energy metabolism (32). Additionally, physical activity stimulates the optimization of mitochondrial biosynthesis and function in the brain, improving the efficiency of cellular energy conversion (33). When sleep abnormalities coincide with low levels of physical activity, metabolic disorders in the brain are exacerbated, leading to inadequate energy support for cognitive activities, which subsequently affects cognitive function (34). Therefore, an integrated approach to regulating sleep and physical activity levels can help maintain cognitive function in older adults through multiple pathways.

Although the present study provides a valuable foundation for understanding the combined effects of sleep duration and physical activity on cognitive impairment, several limitations persist. First, there is the potential for measurement bias in self-reported data. The information regarding sleep duration and physical activity levels was obtained through respondents’ self-reports, which may be influenced by recall bias or subjective perceptual errors. For instance, some older adults may underestimate their actual sleep duration or overestimate the intensity of their physical activity due to memory loss. Future studies should consider incorporating objective monitoring data, such as accelerometers and polysomnography, to accurately record exercise intensity and sleep duration. Additionally, utilizing GPS data could help differentiate between various types of activities, such as walking and housework, thereby enhancing measurement accuracy. Second, the cross-sectional design limits causal inference. The present study, based on the 2020 CHARLS cross-sectional data, was only able to reveal associations between sleep duration, physical activity, and cognitive impairment, but it could not determine the temporal order or causality among these factors. For instance, cognitive impairment may lead to altered sleep patterns or reduced physical activity, and the available data cannot rule out the possibility of reverse causation. In the future, prospective cohort studies should be conducted to track the dynamic changes in sleep, physical activity, and cognitive function in the older adults over an extended period, thereby validating the causal relationships between sleep, physical activity, and cognitive impairment. Additionally, objective measurement tools, such as wearable devices like smart bracelets to monitor sleep duration and quality, as well as motion sensors to accurately record the intensity and duration of physical activity, should be utilized to enhance the accuracy and reliability of the data. Finally, the assessment of cognitive impairment is based on the Brief Mental Status Examination (MMSE) scale, which has limited sensitivity to early mild cognitive impairment and may underestimate the actual incidence of cognitive impairment. It is recommended that future studies incorporate the Montreal Cognitive Assessment Scale (MoCA) to improve the evaluation of dimensions such as attention and executive function.

In summary, the present study, which utilized cross-sectional data from CHARLS, found that both abnormal sleep duration (either short or long) and low levels of physical activity were significantly associated with an increased risk of cognitive impairment in older adults. Furthermore, there was a synergistic effect between the two factors. However, due to the inherent limitations of the cross-sectional design, the findings reflect statistical associations rather than causal relationships. It is recommended that future studies validate the long-term interaction between sleep and physical activity through longitudinal follow-up or experimental interventions. Based on the available evidence, public health authorities should focus on integrated interventions that address both sleep quality and physical activity in older adults. They should fully consider these two lifestyle factors and actively promote joint cognitive-exercise intervention programs to mitigate the risk of cognitive decline.

## Data Availability

Publicly available datasets were analyzed in this study. This data can be found at: https://charls.charlsdata.com/.
